# National registry for sudden unexpected deaths of infants and children in England: why do we need one and do families want one?

**DOI:** 10.1136/archdischild-2018-316542

**Published:** 2019-04-20

**Authors:** Emma Matthews, Peter Blair, Sanjay Sisodiya, Stuart Jones, Neil Sebire, Elijah Behr, Peter Fleming

**Affiliations:** 1 Queen Square Centre for Neuromuscular Diseases, Institute of Neurology, University College London, London, UK; 2 Centre for Child and Adolescent Health, University of Bristol School of Social and Community Medicine, Bristol, Bristol, UK; 3 Clinical and Experimental Epilepsy, University College London Institute of Neurology, London, London, UK; 4 East Surrey Hospital, Redhill, Surrey, UK; 5 Histopathology, Great Ormond Street Hospital For Children NHS Trust, London, London, UK; 6 Cardiology Clinical Academic Group, St George’s University, London, UK; 7 Child and Adolescent Health, University of Bristol, Bristol, UK

**Keywords:** epidemiology, general paediatrics, sids, patient perspective, sudc

## Abstract

The sudden and unexpected death of an infant or child is devastating. An inability to explain why an infant or child died is difficult to accept for both families and professionals. No reliable national dataset exists to estimate precisely how many infants and children die unexpectedly each year in England. This lack of accurate epidemiological data belies the scale of this public health problem. Detailed controlled observational studies of infant deaths identifying risk factors and providing evidence-based advice for parents has seen a dramatic reduction in incidence over the last 30 years by almost 80% but greater knowledge is needed if future deaths of infants and older children are to be prevented and families optimally supported. We propose that a national registry of sudden unexpected deaths in infancy and childhood would accurately determine incidence, identify unknown risk factors and highlight good care practices, ensuring these can be standardised nationally. For such a project to be successful, however, parents must be at the heart of it. We held a consultation day between families, professionals and supporting charities (The Lullaby Trust, Child Bereavement UK, SUDC UK and CRY) to seek opinion on the desire for a registry and how best to ensure families are engaged. Here, we summarise our rationale for a registry and the feedback we received from attendees regarding their views of the proposal and the practical aspects of administering it.

**What is already known on this topic?**

- No reliable national dataset exists in England to determine how many children and infants die unexpectedly each year.
- Previous evidence-based interventions show the value of collecting data systematically and comprehensively.

**What this study adds?**

- There is a need and desire to standardise data collection and care nationally among professionals, families and charities.
- A framework for a national registry and biobank of all sudden and unexpected deaths of infants and children in England is achievable.
- Families showed support for a well-planned registry which engages them and uses robust consent procedures.

## Introduction

While basic information on unexpected deaths of infants and children has been collected since 2008 by Child Death Overview Panels, no reliable national dataset exists to accurately determine how many infants and children die suddenly and unexpectedly without clear aetiological cause in England each year. Such deaths are devastating for families but a lack of accurate epidemiological data compounds efforts to understand why these deaths may occur or how to prevent them. We propose that a national registry of information from investigations and a tissue biobank of samples taken at postmortem examination after sudden unexpected deaths in infancy and childhood (SUDIC) would accurately determine incidence, potentially identify causal and contributory risk factors and highlight good care of bereaved families, for example, sensitive, consistent professional support and signposting families early to appropriate support groups and ensuring these can be standardised nationally.

### Aims and objectives

A registry and biobank of this nature cannot be achieved without parental engagement and consent. We wished to seek opinion from bereaved families as to whether they would support this proposal in principal and to have their insights into what they hoped a registry could achieve. We also wished to ascertain any concerns they had regarding data/tissue analysis and retention and how this may influence consent procedures.

## Methods

We held a 1 day workshop at the Wellcome Collection, London on the 30 April 2018 between parents, charity representatives and professionals to discuss a proposal for a national registry and tissue biobank for all sudden and unexpected deaths of infants and children that occur in England (22 parents, 13 charity and/or physician professionals registered for the event). Bereaved families were invited via charity contacts and from a group who had participated previously in sudden death research. It is recognised this creates a selection bias of families who have already shown a willingness to engage with research. Due to the nature and sensitivity of the discussion however it was felt appropriate to conduct an initial workshop and gain preliminary feedback in this way. The families who attended had suffered the unexpected death of a child from early infancy to 3 years of age. We gave a series of short talks explaining our rationale for such a proposal that included a review of how a lack of accurate epidemiological data limits our knowledge of true incidence and contributory factors to sudden unexpected deaths. We also outlined the current process of investigation when a child dies. This was followed by discussion between all attendees and included predetermined questions. The questions were devised by the authors who felt they represented key pieces of information that were useful to guide the practical conception of a registry. The same questions were provided in a written document to all attendees to write down any additional comments anonymously and 21 completed forms were collected at the end of the meeting.

### Terminology of unexpected deaths

Unexpected infant and childhood deaths (from birth to under the age of 18) are defined as those where the child was not thought by the family or healthcare professionals to be at risk of death 24 hours before the death or the major collapse leading to the death.^1^ Unexpected deaths include those subsequently shown to have occurred from rapidly progressive natural causes (eg, previously unrecognised cardiac abnormalities, or infections), trauma (including accidental and non-accidental injury), drowning and suicide, as well as deaths for which no complete or sufficient explanation can be identified, for example, sudden infant death syndrome (SIDS) <1 year of age, sudden unexplained death in childhood (SUDC) 12 months and older, sudden arrhythmic death syndrome (SADS) or sudden unexpected death in epilepsy (SUDEP). We have used the term SUDIC (Sudden and Unexpected Deaths in Infancy and Childhood) to denote deaths from 0 to 18 years but it was acknowledged in our meeting that the nomenclature and when to use it is confusing and adds an extra layer of complexity for families and professionals.

### Investigation of sudden unexpected deaths and proposal for a registry

The Statutory Guidance to the Children Act 2004 requires that all unexpected deaths of infants and children are subject to a detailed multiagency investigation, including postmortem conducted to an agreed protocol by a paediatric pathologist.^2 3^ All such deaths must be notified to the coroner. Despite thorough investigation including clinical history, scene investigation and autopsy examination being performed, there remain difficulties in objectively determining a cause of death in many cases due to lack of knowledge regarding the clinical significance of some features of the history, circumstances of death and postmortem findings.^4–6^ The Human Tissue Act requires that postmortem tissue samples are disposed of within 3 months of the inquest unless written parental consent is received to retain them for further research and/or teaching and/or future investigations requested by the parents.

The University of Bristol and collaborating partner organisations have recently commenced work, funded by NHS England, to develop a National Child Mortality Database to include all deaths of children from birth to 18 years. Data collected (including data from the child death overview panels), although much more detailed than any currently existing data source, will be limited by the terms of the Children Act 2004, which prevent the use of non-anonymised data collected in this way for research. Such data cannot therefore be linked to the details of the investigations (including postmortem) conducted under the authority of the coroner. Thus, although the Child Mortality Database will be an invaluable resource in identifying patterns of deaths and broad brush assessments of possibly contributory factors, it will not be able to provide an evidence base for risk-reduction advice, or tissue/DNA collection for further investigation or research.

With parental consent, however, the data already collected by the mortality database and any postmortem samples that have been taken, could all be efficiently retrieved and linked for research purposes, avoiding duplication of investigations and optimising the use of time and resources. Additional detailed data could then be collected by research projects with ethical approval, and a registry of tissue samples retained with consent for research.

This would effectively create a national registry of robust consistent clinical, epidemiological and postmortem data, with a federated biobank of tissue samples held with consent for research and provide an evidence base for public health advice. It is proposed research funding be sought for the registry and the administration of data and tissue collections be performed in a joint university/hospital setting.

## Results

### Do families want a national registry?

There was unanimous agreement in the group that the proposition for a registry was an excellent idea. Disbelief was expressed by some families that such a venture did not already exist and concern by others regarding what happens currently to their child’s tissue samples and detailed data they gave at the time of death. It was evident that there were significant variations in the care received by bereaved families and even mandatory standards of care were not always being met. New vision, driven by the imperative of caring for children and their families, was felt to be needed with a proposal for a national registry to ensure 1) standardised, best practice support for families including multidisciplinary review meetings; and 2) international collaboration with global partners to support and undertake coordinated, comprehensive research. The consensus from all attendees was that they would support a well-designed process or registry that had the aim of better understanding why some infants and children die unexpectedly, optimising and standardising care and potentially preventing future deaths.

### What may be the reasons for families not wishing to sign up?

All families indicated that with time following bereavement, they would consent to inclusion in a registry, but the important factors that may influence their response were how, when and by whom they were asked for consent. It was felt to be paramount that the consent process needed to promote signposting to available resources, especially charities who could provide peer support (box 1).

Box 1 **Main outcomes from group discussion and written feedback**

- Families and charities showed support for a national registry of sudden and unexpected deaths in infants and children and biobank.
- Consent must be an extended process with repetition, time to reflect and opportunity for questions.
- Consent must be taken by someone with whom the family has an established relationship and trusts.
- The consent process should be an opportunity to signpost families to support services at an early stage.
- A registry should aim to improve understanding of epidemiological and causative factors and also optimise standards of care of families.
- Participation should be viewed as an opportunity for ongoing two-way engagement in research and service evaluation between professionals and families.
- There was a desire that accessing feedback and outcomes from the registry be a process families could control and do at a time of their choosing.
- There was a desire the registry not be isolated but compatible with other national and international registries.

It is essential that the person asking for consent has a good relationship with the family and is someone they can trust. It was generally felt it was most appropriate that the paediatrician leading the multiagency investigations (required after all such deaths) lead the consent process and take written consent, but it was also suggested bereavement counsellors, nurses, keyworkers and peer supporters or charity befrienders could all provide information about the registry to complement discussions with the paediatrician.

Information regarding the purpose of the registry was also felt to be an important factor associated with success of such an endeavour. There was an acknowledgement from all groups that the registry should be viewed as a long-term project with positive engagement between families and professionals to seek answers and improve care.

### When is the most appropriate time to ask for consent to inclusion in a registry?

Families emphasised that they could recall little or nothing about what was said to them in their first meeting with a paediatrician after their child died. It was generally agreed there should be some interval before the request was made, and the need for multiple discussions was indicated. It was suggested parents could be made aware of the registry in their first meeting with their designated paediatrician, but details discussed at follow-up meetings and again when they met the paediatrician to be informed about the final findings of the child death review process (usually between 3 and 6 months following death), with consent taken at one of the later meetings. It was acknowledged that, although already required under the Statutory Guidance to the Children Act, these meetings do not always take place, are not always conducted by a senior paediatrician and vary in detail.

Families also emphasised that being able to consent and opt in later would be an option people would want (even several years after death). This illustrates a potential need to consider the issue of tissue sample fate and retention separately because of the time limitations imposed on tissue retention currently once the coronial process ceases.

While the process of providing information to families and seeking consent for inclusion in the registry should be explicitly linked to the process of seeking parental views on retention or disposal of tissue samples taken at postmortem examination, it is important that parents are given the opportunity if they wish, to consider the two questions separately.

### How should families receive information about a registry after a child has died?

It was felt critical that parents be given a face-to-face explanation with the opportunity to ask questions and that this be complemented by written material. Clear information was highlighted as a key motivator for families wanting to take part. Charities could also promote the opportunity to take part in a registry, which would be particularly important for families whose children had died before the inception of a registry and for those who may have been unable to consider the possibility at the time of death. There was a suggestion to do exploratory pilot work with families via each charity present, to assess response to receiving information on a proposed registry

### Would families want feedback from a registry and how?

The majority of parents indicated they would like feedback but identified that the nature and timing of this may vary for individuals. There was consensus that for those who agreed to feedback, the ability to access data when parents were ready would be preferable, with suggestions of being given access to a secure website and the ability to log in as they wished, perhaps with an email or written prompt to let them know when updates were available on the website.

### Would families agree to be contacted again for future studies?

There was general agreement by the families that this would be acceptable, although it was emphasised this needs to be explained as part of the informed consent process at recruitment into the registry, with explicit consent for future contact, and that recontact should be from someone with whom the family already have a relationship. Overall, it was felt that the prospect of not merely collecting static data but taking part in future, prospective research was a positive difference between the proposed registry and existing studies. It was recognised not all families would wish to be contacted again. It was stressed there would need to be clarity over the benefit of participation to the wider community but that families may never hear back on an individual basis about the cause of their own child’s death.

### Would families be happy for researchers to publish findings from the registry?

This was supported, as it was felt essential that knowledge be shared as widely as possible. There was also discussion regarding registries in other countries. It was felt paramount that in designing a national registry, there is collaboration with other international groups to ensure data collection was as compatible as possible, enabling pooling of data.

## Discussion

### Estimated incidence of unexpected deaths

More than 4000 children under the age of 18 die every year in England from heterogeneous causes.^7^ No reliable national dataset exists to determine how many of these deaths are sudden and unexpected. Excluding those due to trauma or suicide, the majority of unexpected childhood deaths (we estimate approximately 90%) occur in infants.^8^ There are approximately 250 unexpected infant deaths each year in England (equivalent to a mortality rate of 35 per 100 000 births), of which around 200 remain unexplained.^9^ Most of these meet the definition of SIDS, although guidance from the Chief Coroner incorporated into the Revised Kennedy Report in 20 16^1^ suggests the term ’unascertained’ should be used. In older children, non-trauma-related sudden unexpected deaths are much less common, and their true incidence is unknown. In a case-control study in 2003–2006 from the Southwest Region, we identified between one and three such deaths per year in infants aged 1–2 years, from a population of approximately 60 000 live births per year, suggesting a rate in England of approximately 3 per 100 000 births^10^ (ie, approximately 20 per year). From extrapolation of Child Death Overview Panel (CDOP) data in Southwest England (population 5.5 million), we believe it is likely that at least 20–30 such deaths occur each year in England (population 55 million) in children aged 2–17 years. Although the figures fluctuate year on year, across the last decade the Office of National Statistics reports of unexpected deaths also suggests 20–40 SUDC deaths in England and Wales and 200–400 SUDI deaths occur each year. Although information on all such deaths is collected routinely by CDOPs, there is currently no national aggregation of these data in the UK, and data collected in this way may not be used for research without informed consent from the parents.

### Factors contributing to sudden unexpected deaths

In the late 1980s, >1500 sudden, unexpected, unexplained infant deaths were registered yearly in England. Recognition of the contributory effect to such deaths from the prone sleeping position, and the change in advice to parents nationally in 1991 to place babies on their backs to sleep, was followed by an almost immediate halving of such deaths.^11^

Subsequent case-control studies of other potentially avoidable contributory factors, for example, side sleeping position, hazardous bed-sharing, exposure to tobacco smoke, heavy wrapping and head covering, and the identification of factors that reduce the risk, such as breast feeding, and keeping babies in the same room as parents, led to further advice to parents, followed by a further halving of such deaths over the next two decades.^12^

Sudden unexpected non-trauma-related deaths of older children have been the subject of numerous anecdotal studies, not based on a specified population, that have identified a number of important metabolic and genetically determined underlying conditions, such as fat oxidation defects and cardiac channelopathies,^13^ as well as sudden unexpected death in epilepsy (SUDEP), but in the absence of any systematic data collection on such deaths it is not possible to estimate their frequency or the spectrum of identifiable contributory or causal factors. An increasing proportion of these deaths in the past 20 years have been attributed by coroners to SUDEP, although the evidence on which this label has been based has been very inconsistent, both geographically and temporally.

Overall, it is estimated that around 250 unexpected and unexplained child deaths occur every year in England, but there is a significant gap in accurate epidemiological data and thus our understanding of current potentially contributory or causal factors.^12–14^ Previous evidence-based interventions show the value of collecting data systematically and comprehensively.

## Conclusions

The current relatively low incidence of sudden and unexpected deaths in children aged 1–3 years means that it is not possible to conduct large enough population-based case-control studies within achievable timescales or cost envelopes to further our knowledge and ability to prevent future deaths. Similarly, the low frequency of unexpected deaths among older children has meant that no large-scale population-based studies have been conducted in the UK, and the international studies of such deaths that have been reported suffer from the problems of inconsistencies of investigations, interpretation of findings and attribution of diagnostic labels.^15^

The limitations and inconsistencies in epidemiological data collected, and the lack of aetiological explanation for persisting sudden unexpected childhood deaths, mandate a registry as part of a national service with a global interface. This has been echoed internationally.^16–18^ The sudden unexpected death of a child is beyond tragedy, and the current situation in which we lack explanation for the majority, with fragmented holistic clinical care, is unacceptable to families.

To our knowledge, this is the first report of families’ views on creating a registry for unexpected deaths in infancy and childhood. Individual opinions and wishes will vary beyond our workshop, but there was a consensus that a well-designed collaborative registry with the overall aim of preventing future deaths, and evaluating and implementing good care practices for families, would likely meet with broad support. Useful insights were gained into proposed consent procedures, with opportunities to provide peer and charity support from an early stage. There was positivity towards an endeavour that truly engaged charities, professionals and, most importantly, families.
